# The leaf transcriptome of fennel (*Foeniculum vulgare* Mill.) enables characterization of the t-anethole pathway and the discovery of microsatellites and single-nucleotide variants

**DOI:** 10.1038/s41598-018-28775-2

**Published:** 2018-07-11

**Authors:** Fabio Palumbo, Alessandro Vannozzi, Nicola Vitulo, Margherita Lucchin, Gianni Barcaccia

**Affiliations:** 1 Department of Agronomy, Food, Natural resources, Animals, Environment, University of Padova - Campus di Agripolis, Viale dell’università 16, 35020 Legnaro (PD), Italy; 2 Department of Biotechnology, University of Verona, Strada Le Grazie 15, 37134 Verona, Italy

## Abstract

Fennel is a plant species of both agronomic and pharmaceutical interest that is characterized by a shortage of genetic and molecular data. Taking advantage of NGS technology, we sequenced and annotated the first fennel leaf transcriptome using material from four different lines and two different bioinformatic approaches: *de novo* and genome-guided transcriptome assembly. A reference transcriptome for assembly was produced by combining these two approaches. Among the 79,263 transcripts obtained, 47,775 were annotated using BLASTX analysis performed against the NR protein database subset with 11,853 transcripts representing putative full-length CDS. Bioinformatic analyses revealed 1,011 transcripts encoding transcription factors, mainly from the BHLH, MYB-related, C2H2, MYB, and ERF families, and 6,411 EST-SSR regions. Single-nucleotide variants of SNPs and indels were identified among the 8 samples at a frequency of 0.5 and 0.04 variants per Kb, respectively. Finally, the assembled transcripts were screened to identify genes related to the biosynthesis of t-anethole, a compound well-known for its nutraceutical and medical properties. For each of the 11 genes encoding structural enzymes in the t-anethole biosynthetic pathway, we identified at least one transcript showing a significant match. Overall, our work represents a treasure trove of information exploitable both for marker-assisted breeding and for in-depth studies on thousands of genes, including those involved in t-anethole biosynthesis.

## Introduction

*Foeniculum vulgare* Mill. (2*n* = 2*x* = 22), commonly known as fennel, is a biennial or perennial diploid species belonging to the Apiaceae family (or Umbelliferae, *nomen conservandum*). It originated in the southern Mediterranean and, through naturalization and cultivation, spread all over the world, specifically in dry soils near coastal areas and river banks in Asia, North America and Europe^1^. The wild and cultivated forms of fennel are hermaphroditic; this species of agronomic and pharmaceutical interest reproduces prevalently by outcrossing, but selfing is also possible. Most cultivated varieties are open pollinated (OP), but F1 hybrids have been bred in recent years. Fennel is cultivated both for its inflated leaf bases, which form an edible bulb-like structure that can be eaten raw or cooked, and for its seeds, which are appreciated for their pleasant fragrance and aromatic taste. FAO statistics highlight the economic impact of this species, revealing that India is the world’s leading producer of fennel with more than 500,000 tons per year, followed by Mexico and China^2^.

Fennel is a species of great interest because of its pharmaceutical properties, as it accumulates several compounds with beneficial effects on human health. In this regard, t-anethole is the major component of the essential oils produced by leaves, with a reported content reaching up to 97.1% of the total volatile compounds and with concentrations that vary considerably depending on the phenological state and geographical origin^3^. This organic compound has been extensively explored for its capability in reducing mild spasmodic gastro-intestinal pains^4^ and for its antithrombotic^5^ and hypotensive^6^ activities. From a biological point of view, anethole belongs to the group of phytoalexins, which act as antimicrobials^7^, antifungals^8^, and insecticidals^9^, and it is generally related to plant defence from biotic stresses.

Despite its agronomic and pharmaceutical relevance, the molecular data available for fennel are insufficient, and to the best of our knowledge, few genetic studies have been performed on this species^10^. Most of the genic and genomic DNA sequences currently available in public databases (*e.g*., GenBank) concern the chloroplast genome, whose draft sequence was published together with those of dill (*Anethum graveolens*) and coriander (*Coriandrum sativum*) to highlight the extent of large inverted repeat variation among some taxa of the Apiaceae family^10^.

Until a few years ago, most of the molecular information available to elucidate various complex biological phenomena was derived from extensive investigations on a few model plants. The recent advances in next-generation sequencing (NGS) technologies and the decrease in DNA sequencing costs has led to an increase in the number of transcriptomic studies in non-model plant species^11^. Although the major application of RNA-seq analyses is in the identification of differentially expressed genes (DEGs) amongst different conditions, organs or tissues and developmental stages, this technology is also very useful for the identification of expressed transcripts (ESTs) related to genes involved in metabolic pathways of interest and for the detection of genetic variations such as single nucleotide polymorphisms (SNPs) and simple sequence repeats (SSRs).

In this study, we took advantage of NGS technology to perform the first fennel leaf transcriptome sequencing and *in silico* assembly using two different approaches: *de novo*, without the aid of a sequenced genome, and genome-guided transcriptome assemblies. The first strategy, particularly useful for organisms without a reference genome, is based on the reconstruction of contigs by overlapping the reads obtained from the sequencer based on their high level of redundancy. For species with a reference genome available, a genome-guided assembly is generally preferable. This second strategy aligns reads to a reference genome to finally assemble overlapping alignments into transcripts^12^.

The newly assembled leaf transcriptome dataset will be presented and critically discussed, along with its utility as an important resource for further genetic characterization of cultivated fennel accessions. Specific emphasis will be given to the characterization of the main genes involved in the t-anethole biosynthetic pathway and the identification of single-nucleotide variants exploitable in advanced breeding programmes in *F. vulgare*.

## Results

### Fennel genome sequencing results

Among the 4 lines of cultivated fennel selected in this study, the highly homozygous OL2-2 sample was chosen for genome sequencing (Supplementary Table 1). A total of 486,073,396 paired end reads, corresponding to 72.91 Gbp, were generated using an Illumina HiSeq. 2500 platform (Supplementary Table 2). After the filtering step, we performed a *k-mer* analysis to estimate the overall characteristics of the genome. The 21 *k-mers* spectrum generated by Jellyfish^13^ was analysed with GenomeScope software^14^ to allow a fast reference-free genome profiling from the short reads. The program estimated a haploid genome length of 948 Mb (ranging between 947,35 and 948,76 Mb), an average heterozygosity of 28% (ranging between 26% and 29%) and a repeat content of 52% (Supplementary Table 3). The resulting assembly, obtained using SOAPdenovo2^15^ was highly fragmented and split into several small contigs (7,978,334 contigs, N50 = 319). Considering the assembled scaffolds, we obtained a total number of 300,408 sequences with lengths from 370 to 145,787 bp, for a total of 1.01 Gbp (N50 = 9,443). Table 1 reports the main descriptive statistics related to the genome assembly. To further assess the genome completeness, we performed a BUSCO analysis^16^ using the plant dataset encompassing 1440 genes. Overall, 80.5% of screened genes were identified in the assembled fennel genome. Among these, 73.8% of sequences were complete, with a limited percentage of duplicated genes (5.4%). The remaining 6.7% was composed of fragmented genes (Supplementary Table 4).

**Table 1:** Main descriptive statistics related to fennel genome and transcriptome assemblies.

Main Statistics	Genome	Transcriptome		
		*De novo*	Genome-guided	Clustering
Total sequences	300,408	61,299	51,917	79,263
Total bases	1,011,093,015	57,419,229	68,310,969	90,513,363
Min sequence length	370	104	200	115
Max sequence length	145,787	14,975	13,065	14,975
Average sequence length	3365.73	936.71	1,315.77	1,141.94
Median sequence length	1241.00	627	1,073	824
N25 length	20734	2,313	2,681	2,545
N50 length	9443	1,353	1,850	1,654
N75 length	2842	705	1,162	936
N90 length	1126	425	684	535
N95 length	777	333	471	396
As %	31.81	31.39	29.61	30.62
Ts %	31.72	29.68	30.13	30.06
Gs %	16.06	18.31	21.28	19.76
Cs %	16.11	20.61	18.62	19.3
(A + T)s %	63.53	61.08	59.74	60.68
(G + C)s %	32.17	38.92	39.89	39.06
Ns %	4.30	0	0.37	0.26

Statistics are available for the genome draft, *de novo* transcriptome assembly, the genome-guided transcriptome assembly and the clustered transcriptome assembly.

Finally, trimmed and clean reads were analysed with RepeatExplorer software^17^ to estimate the genome repetitive region content. We allowed the pipeline to automatically select at random the proper number of reads to be processed (884,293). The programs identified 311 clusters. Excluding the 15 clusters associated with organelles (plastid and mitochondria), the repetitive genome content was estimated at approximately 51.8%. The complete list of the characterized repeats is available in Supplementary Table 5. The draft of the Genome Shotgun Assembly project was deposited at DDBJ/EMBL/GenBank under the accession PHNY00000000.

### The first fennel leaf transcriptome assembly

Based on the degree of homozygosity (*i.e*., >90%) estimated using a preliminary screening of 9 hypervariable SSR loci (Supplementary Tables 1 and 6), 4 lines analysed in two biological replicates (namely, OL1-1, OL1-2, OL2-1, OL2-2, OL9-1, OL9-2, OL164-1, and OL164-2) were selected for RNA-seq analysis. A total of 41,189 Mbp, corresponding to 407,850,800 paired-end 101 bp raw reads, was obtained from Illumina mRNA sequencing of *F. vulgare* leaves. Considering the biological replicates for each line, on average 101,962,700 ± 9,372,035 reads were produced (Supplementary Table 2). Raw data were trimmed to remove both the Truseq Universal and Indexed adapters. After removal of low quality sequences, 392,798,001 reads with a final length higher than 25 bp were used for the assembly. Of these, 91.58% reached an average PHRED score threshold of Q ≥30.

Using two different approaches (*de novo* and genome-guided), the reads were first assembled into four distinct transcriptomes, with one for each line. In detail, for the *de novo* approach, we used the CLC Genomics Workbench and Trinity. By comparing the assembly statistics, the output produced by the CLC Genomics Workbench software was superior in terms of N50, number of transcripts and number of assembled bases (Supplementary Table 7), so we adopted it for subsequent analysis. The 4 transcriptomes obtained from the CLC Genomics Workbench software and from the genome-guided approach were further merged to produce two different leaf reference transcriptomes: a *de novo* transcriptome and a genome-guided transcriptome. In this last step, the CLC Genomics Workbench platform enabled us to assemble 61,299 transcripts. The StringTie assembler generated a total of 51,917 transcripts.

A clustering of these two assemblies, performed using CD HIT, was used for subsequent analysis since the number of assembled transcripts and total bases was higher than for the other two assembled transcriptomes considered alone (Table 1). In detail, the “clustered transcriptome” contained up to 79,263 assembled transcripts with an average length of 1,142 bp, N50 length of 1,654 bp and maximal length of 14,975. Overall, 90,513,363 bp were assembled. To assess the completeness of the clustered transcriptome, we performed a BUSCO analysis. A total of 89.1% of the identified genes were complete (19.7% duplicated genes), and 4.8% were fragmented genes, for a total of 93.9% identified genes.

The presence of chimaeras generated during the transcriptome reconstruction and merging process was evaluated using the method suggested by Yang *et al*.^18^. The similarity search against the *Apiaceae* family proteins enabled us to find a hit for 68% of the transcripts. Among these transcripts, the analysis identified only 1,327 transcripts that corresponded to 2.5% chimaera contamination. Finally, the similarity search against a database of plant long non-coding RNA (lncRNAs) enabled us to detect 1,337 lncRNA transcripts. The complete list of lncRNAs identified is available in Supplementary Table 8.

Raw transcriptome sequences files are available on the Sequence Read Archive (SRA) with the accession numbers SSR6265712-SSR6265719. This Transcriptome Shotgun Assembly project was deposited at DDBJ/EMBL/GenBank under the accession GGAC00000000. The version described in this study is the first version, GGAC01000000.

### Functional classification of the clustered transcriptome

BLASTX analysis (E-value ≤ 1e-05) was performed against the Pentapetalae clade subset of the NR protein database and identified up to 47,775 transcripts (60.27% of the total transcript number) showing a significant match. Among these, 8,067 (16.88%) and 4,868 transcripts (10.19%) were related to sequences from *Vitis vinifera* and *Sesamum indicum*, respectively (see Supplementary Table 9). Considering the similarity and E-value distribution, 10,152 (21.25%) assembled sequences showed similarity scores higher than 80%. 19,850 (41.55%) transcripts exhibited extremely low E-values (≤1e-100, Supplementary Tables 10 and 11). Overall, a total of 11,853 (24.81%) transcripts contained a putative full-length CDS, and 2,392 (5.01%) of these revealed similarity scores higher than 80% to their best hit subject.

**Figure 1:**
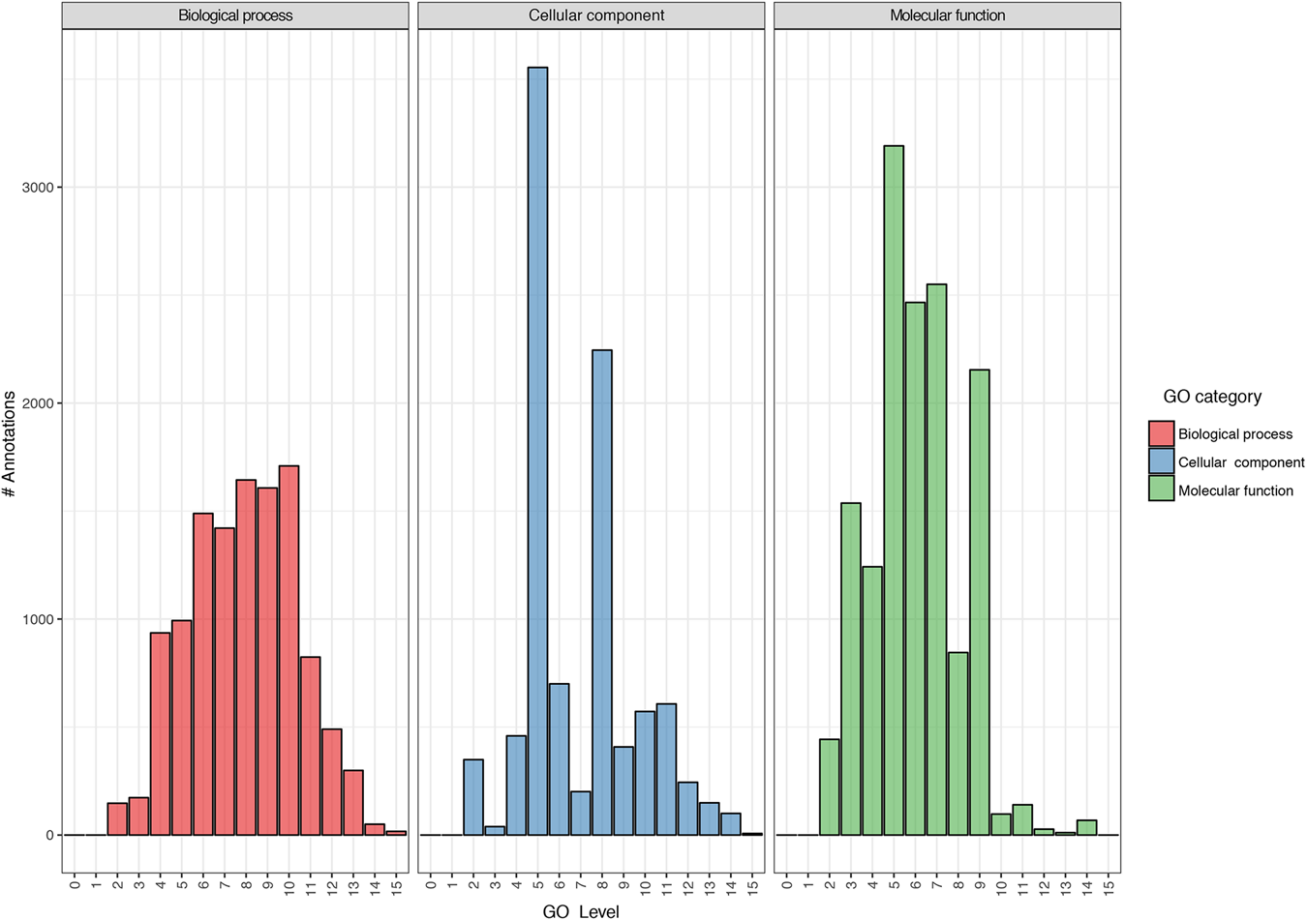
Gene ontology (GO) level distribution chart for the fennel leaf transcriptome according to the Biological Process (BP), Molecular Function (MF) Cellular Component (CC) categories.

The 47,775 fennel transcripts with a BLASTX hit were imported into UniProt for GO mapping and EC annotation. A total of 36,204 GO IDs and 1,629 EC number were assigned respectively to 14,734 and 1,615 fennel transcripts. The GO IDs were distributed in 15 levels among these three categories. Based on number of annotated GO terms, the most informative GO level was level 5, retrieving 7,738 GO IDs (Fig. 1).

**Figure 2:**
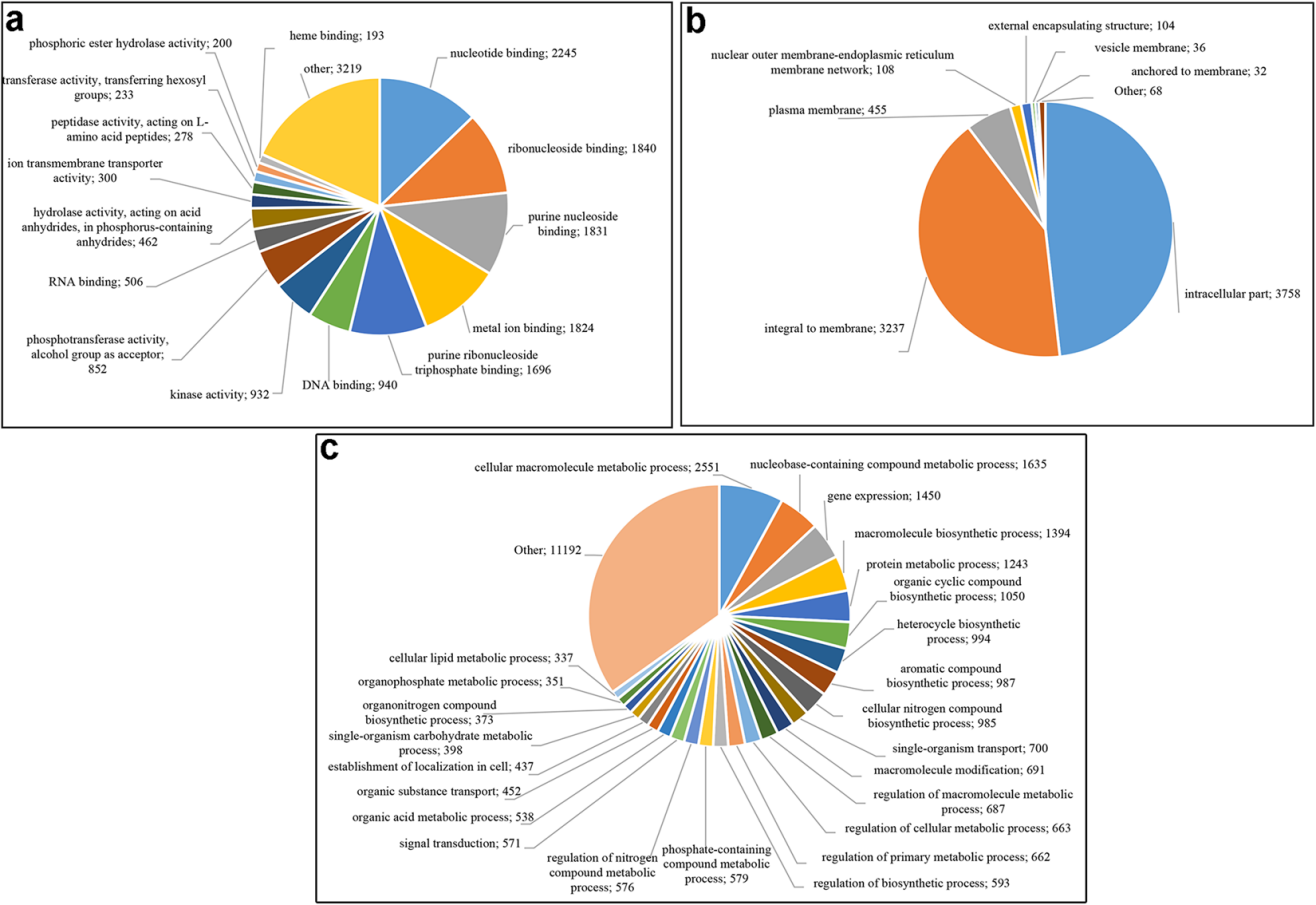
Gene ontology (GO) classification of assembled transcripts. The results of BLASTX searches against the Pentapetalae clade subset of the NR protein database were used for GO term mapping and annotation. The numbers of sequences assigned to level 5 GO terms for GO subcategories including molecular function (**a**), cellular component (**b**), and biological process (**c**), are shown.

Therefore, level 5 was used to summarize the GO terms in subcategories (Fig. 2). In the MF category, “nucleotide binding” (13%), “ribonucleoside binding”, “purine nucleoside binding” and “metal ion binding” (10% each) ontologies were most abundant. Moreover, “intracellular part” (48%) and “integral to membrane” (42%) represented almost all of the CC categories, while “cellular macromolecule metabolic process” (8%), “nucleobase-containing compound metabolic process” and “gene expression” (5%, each) and “macromolecule biosynthetic process” and “protein metabolic process” (both 4%) were the dominant subcategories inside the BP category.

**Figure 3:**
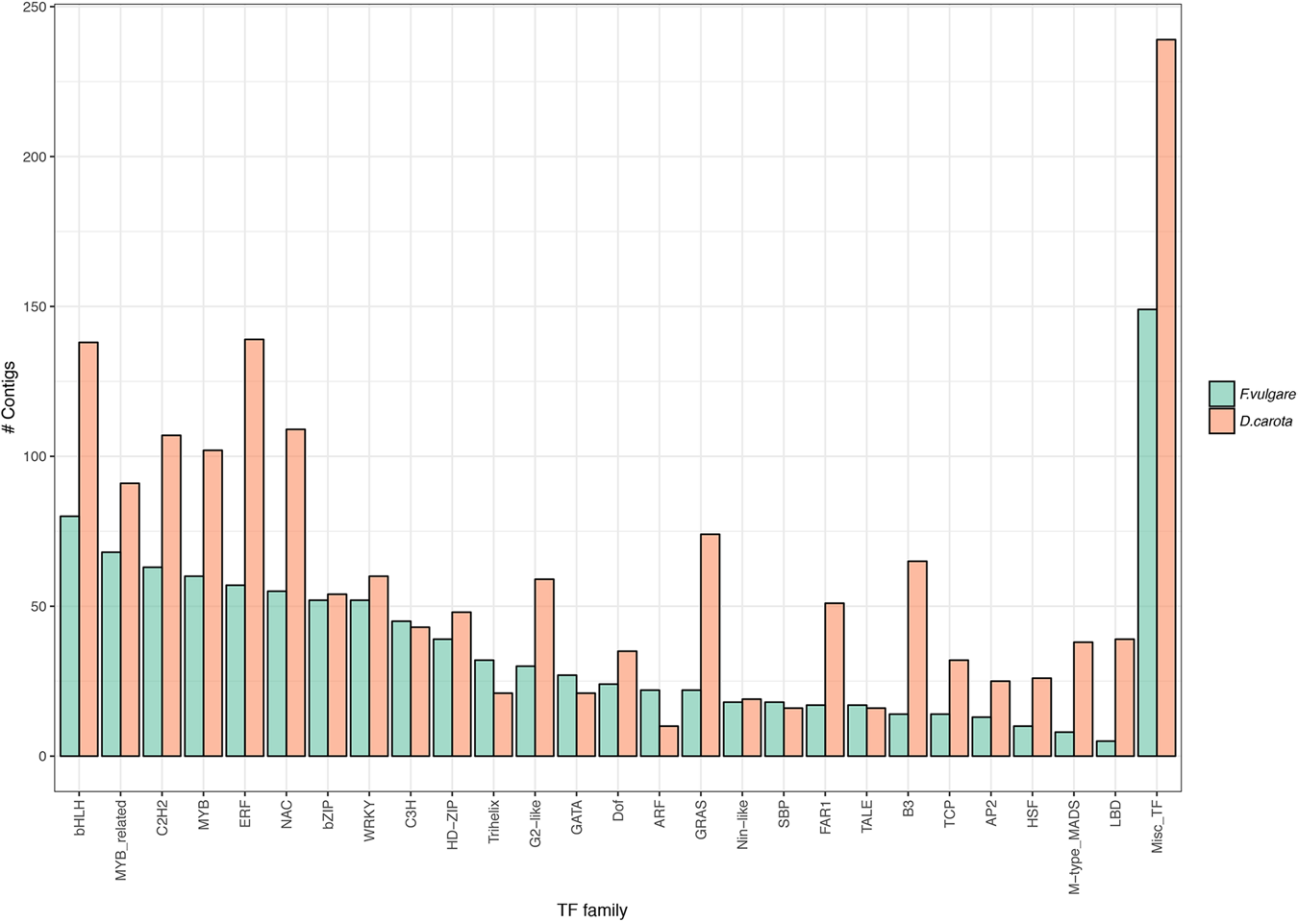
Transcription factor family analysis. The transcription factors determined within the fennel clustered leaf transcriptome assembly are grouped by transcription factor family.

Among the 79,263 transcripts reconstructed in the clustered transcriptome, we identified 1,011 leaf transcripts encoding transcription factors (TFs) based on the plant transcription factor database (PlantTFDB). The abundance of each different multigenic family was evaluated in *F. vulgare* and in *D. carota* (Fig. 3). Among them, BHLH, MYB-related, C2H2, MYB, ERF and NAC were the six most represented categories in both species.

### Identification of gene transcripts involved in the anethole biosynthetic pathway

**Figure 4:**
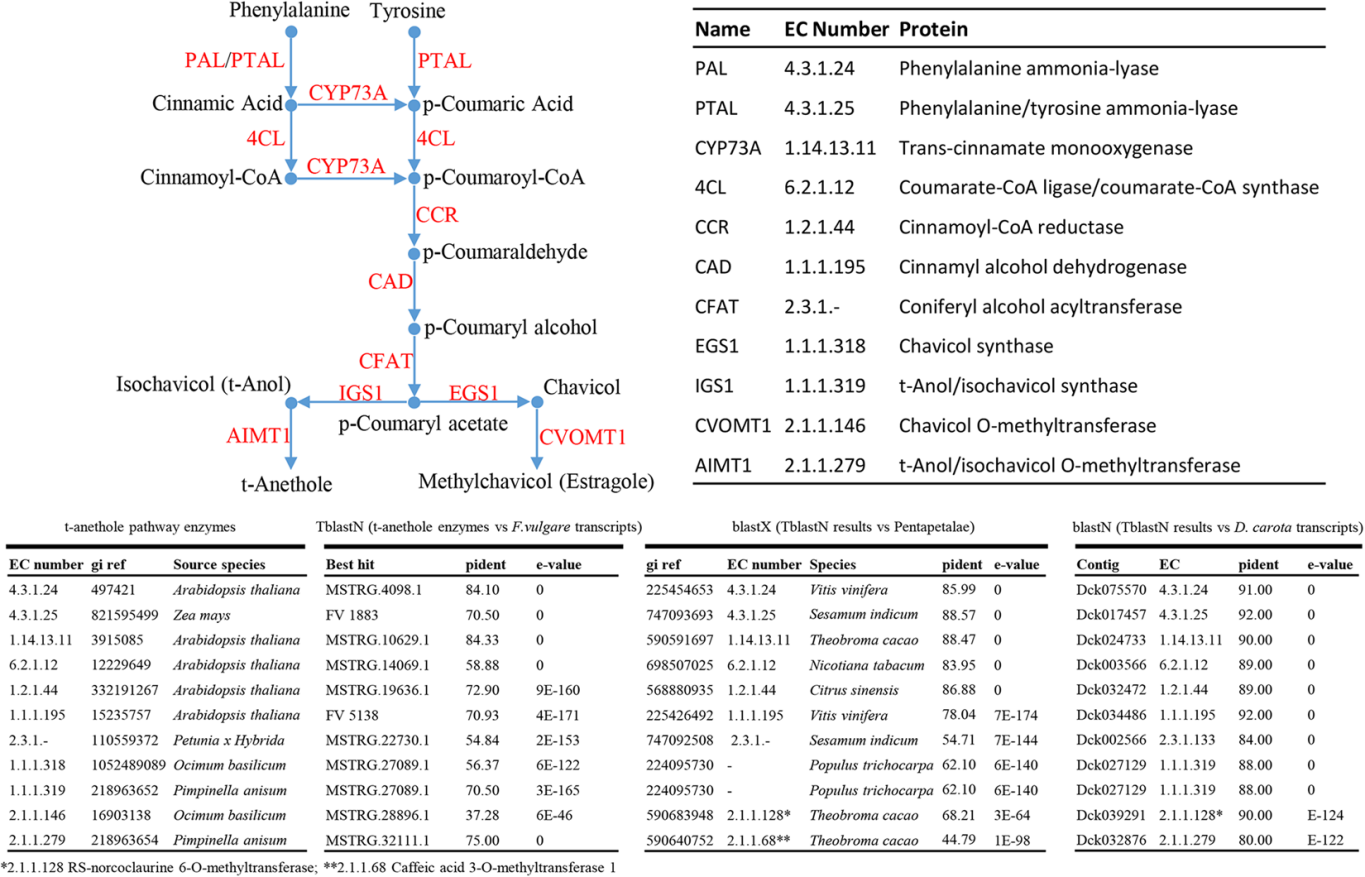
Reconstruction of the t-anethole biosynthetic pathway based on the annotation of the fennel clustered transcriptome assembly. All enzymes involved in the pathway are schematically reported together with their enzyme classification (EC) and abbreviated name. The main BLAST results are summarized in the lower table, including: i) a first TBLASTN approach used to find the best match between the fennel transcriptome and each enzyme of the t-anethole pathway ii) a BLASTX approach used to find the best match between each result of the TBLASTN approach and the Pentapetalae database iii) a BLASTN approach used to find the best match between each result of the TBLASTN approach and the *D. carota* transcriptome.

For each of the 11 protein sequences retrieved from NCBI and involved in the biosynthesis of t-anethole/methyl chavicol, we identified the putative fennel ortholog showing the best significant match (TBLASTN, E-value ≤ 6e-46). These 11 candidate transcripts were successfully aligned to the *D. carota* transcriptome (BLASTN, E-value ≤ 1e-122) and the NR database (BLASTX, E-value ≤ 3e-64). For 7 out of the 11 enzymes involved in t-anethole/methyl chavicol biosynthesis (*i.e*., PAL, PTAL, CYP73A, 4CL, CCR, CAD, CFAT), the results obtained using TBLASTN alignment were coherent also with both BLASTN and BLASTX (Fig. 4). Concerning t-Anol/isochavicol O-methyltransferase (AIMT1, 2.1.1.279), only the BLASTN alignment of the MSTRG.32111 contig against the *D. carota* transcriptome confirmed the results obtained with TBLASTN. Finally, MSTRG.27089 significantly matched (E-value ≤ 6e-122) two different enzymes: chavicol synthase (1.1.1.318) and t-anol/isochavicol synthase (1.1.1.319).

### Identification of expressed EST-SSRs and SNPs

**Figure 5:**
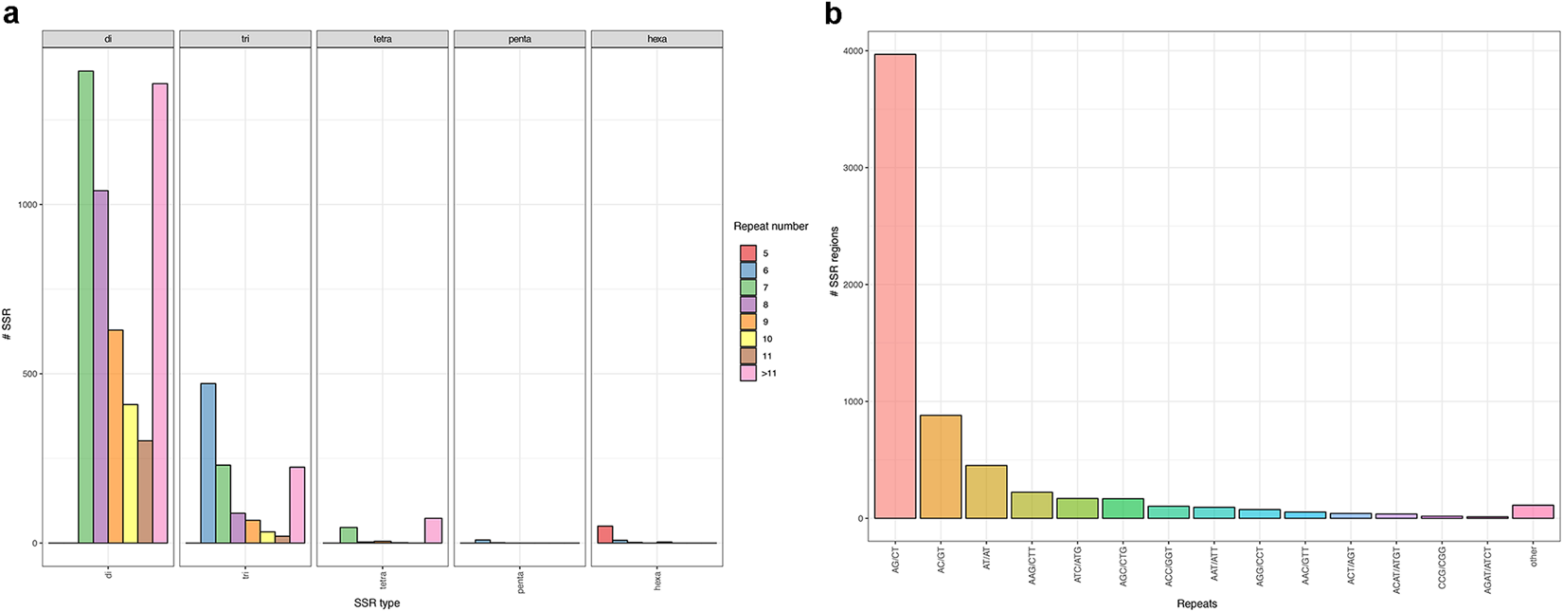
Summary statistics related to EST-SSR regions: (**a**) distribution of the EST-SSR motif repeat numbers from di- to hexa-nucleotide types (the vertical axis shows the abundance of microsatellites with different motif repeat numbers, from 5 to >11, which are discriminated by different colours as reported in the legend); (**b**) most common types of EST-SSR motif repeats.

The MISA program^19^ allowed the identification of 6,411 SSRs in 5,139 transcripts, with 954 sequences containing more than one microsatellite region. Among the SSRs, 4,623 were “perfect”, 9 were “imperfect”, and 1,779 were “compound”. The majority (97.6%) of SSRs were detected in the di- and tri-nucleotide categories (82.7% and 14.6%, respectively), followed by the tetra- (1.2%), hexa- (1%), and penta-nucleotide categories (0.1%). The results of the EST-SSRs are summarized in Fig. 5. The most common dinucleotide was AG/CT (64.9%), followed by AC/GT (13.7%).

43,237 SNPs and 3,955 indels were also identified among four lines analysed in two biological replicates by applying a SNP calling approach based on deep multiple alignment (minimum 8× coverage) and allowing no more than two missing data points. The global inter-sample SNP frequency was 0.5 per Kb, and the indel frequency was 0.04 per Kb. The transition/transversion ratio was 1.65.

**Figure 6:**
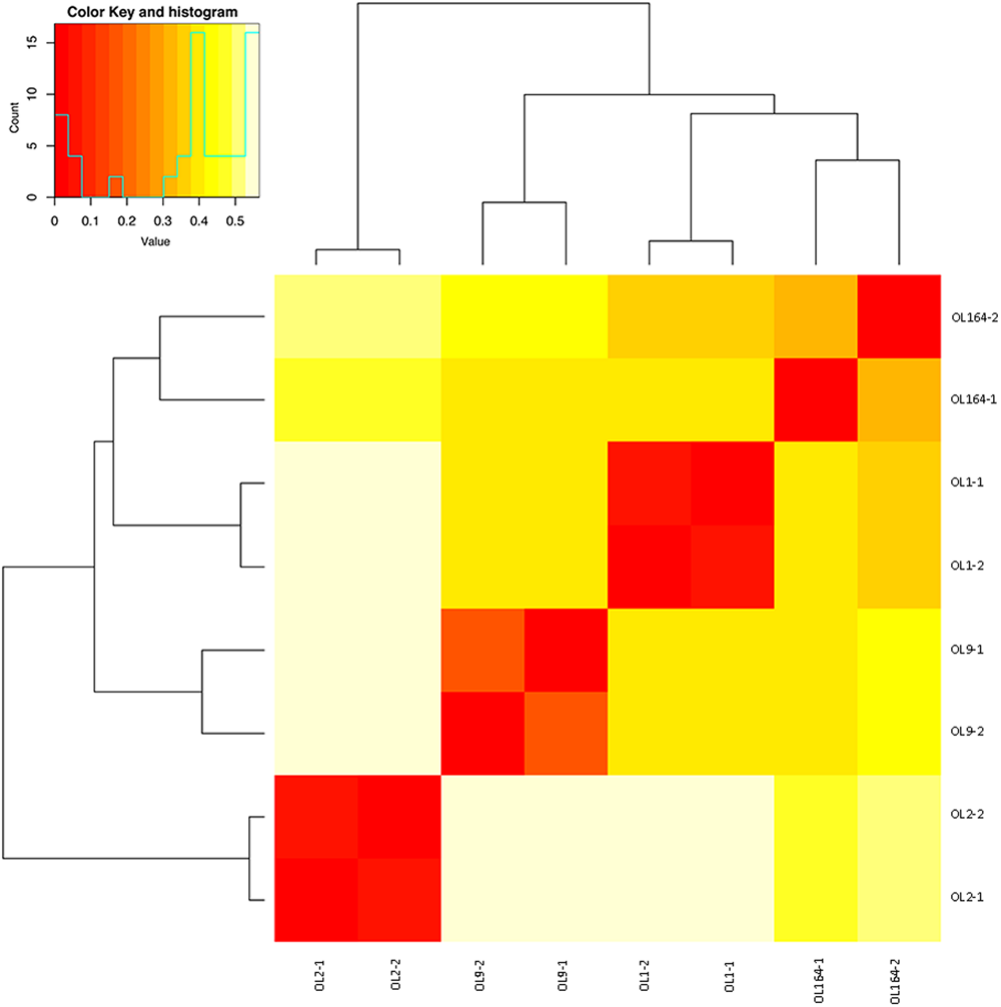
Heat map representing genetic similarity as calculated using the simple matching coefficient in all possible pairwise comparisons based on SNPs identified in the 8 individuals (belonging to 4 breeding lines analysed in two biological replicates) under study.

In more detail, the biological replicates belonging to OL1 and OL2 showed an intra-line similarity close to 100%. OL1-1 and OL1-2 shared 99.06% of SNPs, while the percentage of common SNVs detected between OL2-1 and OL2-2 was even higher (99.27%). On the other hand, the intra-line similarity was considerably lower for OL161 (71.65% of SNPs shared between OL161-1 and OL161-2) and OL9 (79.70% of SNPs shared between OL9-1 and OL9-2). The genetic distance between individuals belonging to different lines varied from 0.35 (OL1 vs. OL164) to 0.57 (OL9 vs. OL2), as reported in Fig. 6. Finally, the SNP calling analysis enabled us to determine the degree of homozygosity for each sample. It ranged from a minimum of 67% (OL1-1) to a maximum of 84% (OL2-2). On average, the homozygosity was 74%.

## Discussion

The technological revolution in NGS brought unprecedented opportunities to study any organism of interest at the genomic and transcriptomic levels. RNA-seq allows us to deep screen the entire transcriptome, which includes all expressed sequences and represents a reduced representation of the genome^20^. Despite the agronomic and pharmaceutical relevance of *Foeniculum vulgare*, the available molecular data on fennel are limited, and only a few genetic studies have been performed in this species so far.

With this aim, we used NGS technology to develop a draft genome assembly and leaf transcriptome sequencing using two different transcriptome reconstruction strategies: one without the aid of a reference genome (*de novo* assembly) and one based on the newly developed reference genome (reference genome-guided assembly).

As expected, the draft genome assembly was highly fragmented (300,408 scaffolds). Although the complete assembly of the fennel genome is beyond the scope of the current work, it is worth mentioning that this preliminary clustering enabled us to assemble 1.01 Gbp that, in agreement with the result of the *k-mer* analysis (0.95 Gbp), represents approximately 70–73% of the estimated genome size based on flow cytometry^21^ (1 C = 1.37 Gbp). Moreover, the BUSCO analysis^16^ confirmed that more than 80% of the genes (considering both complete and fragmented sequences) were included in the assembly, suggesting that the vast majority of coding sequences were correctly represented in the genomic scaffolds and that this assembly is a reliable draft for the genome-guided assembly of the leaf transcriptome.

The repetitive element content of the fennel genome, which was estimated using RepeatExplorer, was 51.8% and agrees with the estimate determined using the *k-mer* analysis (52% repetitive content). From a comparison with the carrot genome, the only other species from the Apiaceae family for which a genome is available, the number of repetitive elements detected in fennel was slightly higher (52% vs 46%). This finding should correlate with huge differences in genome size between these two species. In fact, the estimated haploid genome size of *Foeniculum vulgare* is about three times larger than that of *Daucus carota* (1.37 Gbp and 0.473 Gbp, respectively), and it is widely accepted that a variation in genome size is closely correlated with the amount of repetitive DNA^22^.

In regard to the leaf transcriptome assembly, we decided to implement two different strategies to highlight their advantages and disadvantages. In general, *de novo* transcriptome assembly is much more computationally consuming than genome-guided assembly. It is used when reference genomes are not available but can also be utilized when genome references are available. In our strategy, we considered both a *de novo* assembly based on transcriptome sequencing (RNA-seq) conducted on four economically relevant lines of *F. vulgare*, namely, OL1, OL2, OL9 and OL164, and a genome-guided assembly performed using a low-coverage draft reference genome obtained from whole-genome sequencing of the OL2 line.

As expected, *de novo* transcriptome assembly produced a higher number of contigs (61,299 transcripts) compared to the *ex post* genome-guided assembly (51,917 transcripts). The difference between the average transcript length of the latter (1,027 bp) and the former (627 bp) suggests a conspicuous fragmentation of the *de novo* assembled-transcripts into multiple pieces. Moreover, the lower number of transcripts produced by the genome-guided assembly could be due to the partial coverage of whole-genome sequencing. Consequently, mapping of RNA-seq reads on short and partial genomic contigs allowed us to obtain only a partial reconstruction of the fennel transcriptome. Conversely, the *de novo* transcriptome assembly, being independent of the reference genome, could reconstruct transcripts that were not in the reference due to missing portions of the genome, structural variants or other reasons^23^. Although genome-guided assemblies still have merits as *ex post* transcriptome assembly, limitations imposed by spliced alignments, such as errors and artefacts, can negatively influence the results^12^. To achieve a higher level of reliability for the fennel transcriptome to obtain a more comprehensive transcriptome, we attempted a combined approach by combining the genome-guided and *de novo* assemblies into a clustered transcriptome. This approach led to a transcriptome with several transcripts higher compared to both the other approaches (79,263) and with an intermediate average transcript length (1,141.94). The completeness of the clustered transcriptome was very high because, as indicated by the BUSCO analysis, a total of 93.9% genes (both complete and fragmented) were detected in the transcriptome. Another parameter that proved the good quality of the assembly is the almost total absence of chimaeras generated during the transcriptome reconstruction and merging process. According to the method suggested by Yang *et al*.^18^, the percentage of chimeric transcripts was extremely low (2.5%). Focusing on the clustered transcriptome, we could annotate 47,775 (60.27%) transcripts, and a large percentage of them were related to sequences from grapevine (16.88%) and sesame (10.18%). Of the 47,775 transcripts with BLASTX hits, the GO analysis determined GO IDs and enzyme code (EC) assignments for 14,734 (30.8%) with full or partial annotations (see Fig. 1). Of the 14,734 annotated transcripts, 1,615 have predicted functions (EC codes). Cellular metabolic processes were among the most highly represented groups in terms of GO analysis. This was expected given that young leaves are undergoing rapid growth and extensive metabolic activities.

Considering the pivotal role of transcription factors in regulating many plant processes and functions, we focused on this category. We identified 1,011 transcripts, corresponding to 2.1% of the total annotated transcripts identified. This result is totally in agreement with observations in *Daucus carota* from the same family, Apiaceae^24^: in 57,128 annotated transcripts, 2.9% of them (1,677) matched with as many transcription factors. The overall distribution of transcription factors in fennel within the known TF families is similar to observations in other species like winged bean^25^, chickpea^26^ and, carrot^24^. The TF family most represented in our data was the bHLH, a superfamily of TFs representing important regulatory components in many transcriptional complexes and controlling processes like the regulation of flavonoid biosynthesis, epidermal cell fate determination like stomata formation, hormone response and light signalling^27,28^. Together with bHLH, the most enriched TFs in our analyses belonged to the C2H2, ERF, NAC, MYB and MYB-related families. Surprisingly, these families are also the six most represented families in *D. carota* (see Fig. 3). All the abovementioned TFs are organized in large gene families in plants, with numbers comparable to those observed in our study. However, the low coverage of our assembly and the partial annotation of sequences make it difficult to compare.

Simple sequence repeats (SSRs), or microsatellites, are widely used for genetic diversity analyses and marker-assisted breeding programmes because of their highly polymorphic and discriminant nature, co-dominant inheritance, prevalence throughout the genome, ease of use and cost-effectiveness. Being located in the coding regions, expressed SSRs (EST-SSRs) have increased amplification success in related species, and they are useful for assessing functional diversity and for marker-assisted selection^29^. Moreover, they can act as anchor markers for evolutionary and comparative mapping studies due to their higher transferability among related species compared to genomic SSRs^30–32^. As a counterpart, expressed SSRs usually possess a lower level of marker polymorphism than do genomic SSR markers^33–35^. The screening of the assembled transcriptome led us to identify 6,411 microsatellite regions, most belonging to the di- and tri-nucleotide category (for details see Fig. 5). This finding is similar to reports in celery^36^, sesame^37^, peach^38^, kiwi fruit^39^, and rice^40^. On the other hand, tetra-nucleotide repeats dominate in species such as bread wheat^41^, grapevine^42^ or sugarcane^30^.

Together with the EST-SSRs, we screened our transcriptome assemblies for single nucleotide polymorphisms (SNPs), another class of markers that can help assess genetic variation, are less polymorphic than SSRs, abundant, and easily obtained using NGS. In our study, we identified approximately 43,000 SNPs and 4,000 indels in the 8 tested samples. The informativeness of this new set of SNPs was successfully evaluated by calculating the genetic distances in pairwise comparisons among the 8 individuals. As expected, genetic distances between individuals from different lines were always higher (>0.35) than those drawn from samples belonging to the same lines (<0.30). In more detail, it is worth highlighting some aspects: (i) marked dissimilarities among the lines (*e.g*., OL9 vs OL2) support the possibility of proceeding with crossing schemes to develop highly heterozygous F1 hybrids or to increase segregation potentials in F2 progenies; (ii) significant differences between individuals belonging to the same line (*e.g*., between OL164-1 and OL164-2) could unveil accidental pollinations and genetic contaminations; (iii) low homozygosity (*e.g*., OL1-1 and OL1-2) suggests that additional cycles of selfing should be performed to achieve higher genetic uniformity of the lines. Although statistics from SNP calling analysis cannot be compared with those from SSR genotyping analysis (>40,000 SNPs vs 9 SSRs), OL2-2 was the most homozygous individual with both marker systems. Moreover, the average level of heterozygosity calculated from the SNP analysis (26%) was consistent with the mean heterozygosity calculated by GenomeScope (28%) through *k-mer* spectrum analysis. Validation of the identified SNPs was not the scope of this study, but we believe that this inventory of nucleotide variants presents a significant resource for future work in plant breeding activities and genetic diversity studies. These are the first SNP markers discovered in fennel. These markers could be used in both Mendelian gene and quantitative trait loci (QTL) mapping, generating genetic linkage maps, genotyping and breeding programmes. The frequency of SNPs and indels were 1 SNP every 2 Kb and 1 indel every 25 Kb. The frequency of single-nucleotide variants, such as SNPs and indels, is supposed to be lower in transcribed regions compared to non-coding regions within the genome. Finally, the transition:transversion ratio was equal to 1.65. Similar ratios were found across SNPs in other Apiaceae species, such as carrot, where the ratio of transition substitutions was approximately 1.75 to 1^43^.

The available scientific research on fennel revealed that it is an important medicinal plant used in a wide range of ethnomedical treatments, including for abdominal pains, vomiting, arthritis, cancer, diarrhoea, and others^44^. Moreover, studies carried out in the past indicate that fennel possesses diverse health benefits. Extracts of fennel possess a range of pharmacological actions, such as antiaging, antiallergic, anticolitic, antihirsutism, anti-inflammatory, antimicrobial and antiviral activities^44^. Among the large number of chemical constituents identified in fennel, the volatile component t-anethole probably represents the most important, both in terms of its organoleptic effect, conferring the typical anise taste, and in terms of its medical role. The biosynthesis of t-anethole was recently elucidated by Koeduca *et al*.^45^, who identified and characterized two genes encoding t-anol/isoeugenol synthase 1 (IGS1) and t-anol/isoeugenol O-methyltransferase 1 (AIMT1) in *Pimpinella anisum*. It is known that IGS1 uses coumaroyl acetate substrate to catalyse the formation of t-anol, and AIMT1 catalyses the formation of t-anethole through a methylation step. The screening of the 47,775 annotated transcripts from the clustered transcriptome allowed us to identify all genes belonging to the pathway (including PAL, PTAL, CYP73A, 4CL, CCR, CAD and CFAT, as shown in Fig. 4) involved in the biosynthesis of the coumaroyl acetate. Moreover, we identified two transcripts encoding genes putatively involved in the t-anethole biosynthesis. The first transcript (MSTRG.27089.1) significantly matched (E-value 3e-165, similarity 70.5%) with the t-anol/isochavicol synthase (IGS1, EC: 1.1.1.319) characterized in *P. anisum*, (gi|218963652), and the second one (MSTRG.32111.1) probably encoded (E-value 0, similarity 75.0%) the t-anol/isochavicol O-methyltransferase (AIMT1, EC: 2.1.1.279) described by Koeduca *et al*. (gi|218963654). Considering the close relationship between structural genes involved in the parallel pathways leading to t-anethole and methyl chavicol (estragole) biosynthesis, we were not able to discriminate whether our transcript encodes structural genes involved in this pathway rather than the other based on the sequence identity. Although it has been shown that the amount of estragole and trans-anethole produced by *F. vulgare* varied consistently during plant development^1^, the production of t-anethole was very high^3,46^ compared to the accumulation of estragole in this species. Taking into account this aspect, it is reasonable to think that the two transcripts may encode structural genes involved in the t-anethole pathway.

In conclusion, the newly assembled leaf transcriptome will represent an innovative knowledge step and valuable molecular dataset exploitable for the genetic and functional characterization of wild and cultivated fennel materials. We are confident that the bioinformatic characterization of the main genes and gene products involved in the t-anethole biosynthetic pathway and the identification of single-nucleotide variants will soon be useful for breeding new varieties of *F. vulgare*.

## Materials and Methods

### Plant material

Plant materials used in this study were selected based on the following criteria: (i) commercial importance of the variety to which each line belongs; (ii) robust phenotypic characterization available for each breeding line; (iii) representativeness of cultivated biotypes showing distinct esthetical, agronomic and aromatic traits as well as unrelated genetic backgrounds; (iv) high degree of homozygosity (>90%). Regarding this latter parameter, a small set of 9 SSR markers, developed *ad hoc* both from inter-microsatellite and microsatellite DNA regions isolated in *Daucus carota*, was used to preliminarily examine and characterize a core collection of 96 samples belonging to 10 different breeding lines (Supplementary Table 6). Four breeding lines, namely OL1, OL2, OL9 and OL164, in two biological replicates, resulted suitable for the purpose of the analysis and were registered in the NCBI Sequence Read Archive with biosample accession numbers SAMN07983890- SAMN07983894, SAMN07983897- SAMN07983899. Samples are available upon request. Seeds were sown and plants grown in greenhouse, under light/dark cycle conditions of 16/8 h and temperature of 22–24 °C. Leaves were collected from 1-month-old individuals, snap-frozen in liquid nitrogen upon harvesting and stored at −80 °C until further processing.

### DNA/RNA isolation and sequencing

Among the eight individuals considered, OL2-2, being characterized by the highest degree of homozygosity (93%) was employed for genome sequencing. Genomic DNA was extracted from leaf tissues using a standard CTAB protocol^47^. The quality of gDNA was estimated by spectrophotometric analysis (NanoDrop 2000c UV-Vis, Thermo Fisher Scientific) and agarose gel electrophoresis (1.0% w/v agarose TAE 1× gel containing 1× SYBR® Safe, Thermo Fisher Scientific). A total of 2 µg of genomic DNA were subjected to library preparation for whole-genome sequencing using the Illumina TruSeq DNA PCR-free sample preparation kit (Illumina, Inc., San Diego, CA, USA) with an insert size of ~350 bp. The library was sequenced on a lane Illumina HiSeq. 2500 using paired-end, 150-bp-read chemistry (Illumina, Inc., San Diego, CA, USA).

Total RNA was extracted from the eight samples above mentioned (4 lines in two biological replicates) using RNeasy Plant Mini Kit (Qiagen GmbH, Hilden, Germany), and treated with RNase-Free DNase set (Qiagen GmbH, Hilden, Germany) according to manufacturer’s instructions. The quality of the RNA samples was evaluated by spectrophotometric analysis and agarose gel electrophoresis. In addition, the integrity was analyzed using the RNA 6000 Pico Kit (Agilent Technologies, Santa Clara, CA) on a Bioanalyzer 2100 (Agilent Technologies). Samples with RIN (RNA Integrity Number) values of at least 7 were considered suitable for the following steps.

An equal amount of total RNA (1 µg) from each of the 8 samples was used as input for the TruSeq Stranded mRNA LT Sample Prep Kit (Illumina, Inc., San Diego, CA, USA) and, by means of indexed adapters, a sequencing library was created according to the manufacturer’s instructions, with an insert size of ~300 bp. The library was then sequenced on a lane Illumina HiSeq. 2000 (Illumina, Inc., San Diego, CA, USA) with paired-end, 100-bp-read chemistry.

### Genome draft assembly

Raw genomic sequences were processed with Trimmomatic software^48^ to remove the adapter sequences and to trim low quality bases. In particular, Trimmomatic was run setting an average minimum quality score of 20 within a sliding window of 5 and the minimum reads length was set to 75 bp. The filtered sequences were firstly used to perform a k-mers analysis in order to estimate the overall characteristic of the genome. The analysis was performed using the “count” module implemented in the Jellyfish software^13^ and setting the length of the k-mers to 21. The output of this program was then converted into an histogram (*histo* module from jellyfish package) and provided as an input to GenomeScope software^14^. GenomeScope is a free-reference, open-source web tool to rapidly estimate the overall characteristics of a genome such as genome size, heterozygosity rate and repeat content from unprocessed short reads (http://qb.cshl.edu/genomescope/). The program was run using default parameters setting the max *k-mers* coverage to 2000.

The filtered sequences were assembled using SOAPdenovo2^15^ into contigs using a multi k-mers approach (progressive *k-mers* from 71 to 121 with steps of 1). Sequence statistics were calculated using a Perl script, NGSQCToolkit_v2.3.3^49^: total number of reads/sequences, total and individual (A, T, C, G and N) number of bases, G + C and A + T counts, and minimum, maximum, average, median, N25, N50, N75, N90 and N95 length N50 value, were evaluated. In order to validate the completeness of the reconstructed genome and transcriptome we performed an analysis with BUSCO software^16^. BUSCO was run with the embriophita db dataset that contains 1,440 near universal single copy orthologs.

The repetitive sequences content of the genome was finally assessed by means of RepeatExplorer software^17^. The program was run through the Galaxy portal (https://repeatexplorer-elixir.cerit-sc.cz/) using default parameters and grouping the preprocessed genomic reads into repeat clusters.

### Leaf transcriptome assembly

Raw RNA sequence data were filtered using standard RNA-Seq parameters by means of CLC Genomics Workbench 7.0.4 (CLCbio, Aarhus, Denmark). Briefly, raw reads were demultiplexed and the 3′ ends were trimmed to form eight sets of reads from the four different lines (each one in two biological replicate). Reads were then processed as follows for: (i) removing low quality sequences with a 0.05 error probability limit; (ii) discarding reads with final length <25 bp; (iii) trimming reads with more than two ambiguous nucleotides. Two different bioinformatics approaches, a *de novo* and a genome-guided transcriptome assembly, were then evaluated independently.

In the first approach, each line (comprising reads from two biological replicates) was *de novo* assembled separately using CLC Genomics Workbench 7.0.4 (CLCbio, Aarhus, Denmark), run at default settings. Through the “*de novo* assembly” option, the four transcriptome assemblies (one for each line) were, in turn, assembled into a global one, considered as a *de novo* leaf reference transcriptome. Assembly statistics of the output (i.e. four transcriptomes and global transcriptome) produced with CLC Genomics Workbench were compared to those ones related to an alternative *de novo* assembly carried out by means of Trinity (trinityrnaseq_r2013-02-25) software^50^ (run at default settings), being one of the most popular tools for this kind of analysis e.g.^51–54^.

For the *ex post* genome-guided assembly, filtered RNA-seq data from the two biological replicates of each line were aligned against the draft reference genome newly developed using HISAT^55^ at default settings. Afterward, StringTie^56^ was first used to reconstruct the transcriptome of each line and finally to collapse them into a global genome-guided leaf reference transcriptome, using the “merge” option.

A final clustering was accomplished overlapping the two newly assembled *de novo* and genome-guided transcriptomes: CD-HIT^57^ was used to cluster all sequences with a similarity threshold >95% and to generate a third transcriptome designed as “clustered transcriptome”. CDHIT algorithm performs a clustering based on an identity threshold. For each generated cluster (group of sequences that share the identity threshold), CDHIT returns the longest sequence as a representative of the cluster. The quality of the three transcriptome assemblies (*de novo*, genome-guided and clustered transcriptomes) was then assessed using NGSQCToolkit_v2.3.3^49^ and through a BUSCO^16^ analysis, as already did for the genome draft.

To assess the presence of chimaera sequences generated during the assembly and merging process, we used the methods as suggested by Yang *et al*.^18^. All the programs and guideline are available at https://bitbucket.org/yangya/optimize_assembler. At first, we performed a similarity search against a proteome of closely related species (*Apiaceae* family), using BLASTX and setting an e-value cutoff of 0.01. The blast output file was then parsed and analyzed using the program “detect_chimera_from_blastx.py”.

Finally we also performed a similarity search against a databases of plant long non coding RNA (lncRNAs). The database was downloaded from CANTATAdb 2.0^58^, and contains 239,631 annotated lncRNAs in 36 different plant species and 3 algae. We performed a similarity search using BLASTN program and setting an e-value cutoff of 1-e20.

### Functional annotation and classification

The clustered transcriptome was validated by means of a comparison with a subset of the NR protein database focused on the Pentapetalae clade, using a BLASTX-based approach (E-value ≤ 1e-05, BLAST v.2.3.0+). Assumed that each assembled transcript represented a single gene, the best hit for each transcript was selected. Moreover, in order to extrapolate Gene Ontology annotations^59^ and KEGG terms^60^, the GI identifiers of the BLASTX hits were mapped to the UniprotKB protein database^61^. In addition, a sequence was predicted as full-length transcript if the ratio between its BLASTX alignment length and the subject length extrapolated from UniprotKB protein database was higher than 0.95.

Finally, further enrichment of enzyme annotations was made with the BLAST2GO software v1.3.3^62^ using the function “direct GO to Enzyme annotation” to perform basic statistics on ontological annotations, reducing the complexity of the data.

PlantTFDB^63^, a comprehensive database of transcription factors (TFs) from 165 species, was used to translate scaffolds from the clustered transcriptome to protein and to predict TFs *in silico* (E-value ≤ 1e-05). Through the “Best hit in *Arabidopsis thaliana*” tool, it was also possible to detect the best match between each transcription factor predicted in fennel and the *Arabidopsis thaliana* TF database. The results were finally compared with the TF abundance in the transcriptome of *D. carota*, from the same Apiaceae family.

### Characterization of the t-anethole and methyl chavicol biosynthetic pathways

For the identification of transcripts related to the t-anethole/methyl chavicol biosynthesis, 11 amino acid sequences associated to enzymes functionally characterized and known to be involved in these pathways were retrieved from NCBI. Sequences were used as query in a TBLASTN-based approach (E-value ≤ 1e-20) against the clustered transcriptome. Finally, to confirm or discard those matches found in the first TBLASTN approach, a reciprocal BLASTX approach was carried out using the clustered transcriptome as query and the amino acid sequences retrieved from NCBI as database.

Those transcripts that confirmed the match in both the alignments were considered putative orthologous and were, in turn, aligned against: *Daucus carota* transcriptome^24^, which belongs to the same family (Apiaceae, BLASTN, E-value ≤ 1e-50) and against the Pentapetalae clade subset of the NR protein database (BLASTX, E-value ≤ 3e-50) to further confirm the previous results.

### Simple sequence repeats (SSRs) identification

Simple sequence repeat regions were detected using the MIcroSAtellite (MISA) Identification Tool Perl script^19^. The clustered transcriptome was screened for di-, tri-, tetra-, penta- and hexa-nucleotide repeat motifs with a minimum repeat number of 7, 6, 6, 6 and 5, respectively. The maximal number of nucleotides interrupting two SSR regions in a compound microsatellite was set at 100 bp and the space between imperfect SSR stretches was set at 5 bp.

### Single nucleotide polymorphisms (SNPs) identification

The transcriptome reads were processed for adapter removal, quality trimming and filtering for organelle DNA and duplicates. Post-processed paired-end reads longer than 50 bp were aligned to the clustered reference transcriptome using BWA^64^ with default parameters. Local realignment around In/Dels was performed with the RealignerTargetCreator and IndelRealigner tools of the GATK package, version 2.1-13^65^. Variant positions were identified using the HaplotypeCaller tool of the GATK package with default parameters. Depth-of-coverage was analyzed using DNAcopy. SNP variant were hard filtered using the VariantFiltration tool according to GATK instruction. The pairwise genetic similarity (dissimilarity) among the 8 individuals (4 breeding lines in two biological replicates) was estimated based on the simple matching genetic similarity coefficient using NTSYS software (http://www.exetersoftware.com/cat/ntsyspc/ntsyspc.html).

## Electronic supplementary material

Supplementary Table 1

Supplementary Table 2

Supplementary Table 3

Supplementary Table 4

Supplementary Table 5

Supplementary Table 6

Supplementary Table 7

Supplementary Table 8

Supplementary Table 9

Supplementary Table 10

Supplementary Table 11
